# New ribotype *Clostridioides difficile* from ST11 group revealed higher pathogenic ability than RT078

**DOI:** 10.1080/22221751.2021.1900748

**Published:** 2021-04-05

**Authors:** Wenpeng Gu, Wenguang Wang, Wenge Li, Na Li, Yuanyuan Wang, Wenzhu Zhang, Caixia Lu, Pinfen Tong, Yuanyuan Han, Xiaomei Sun, Jinxing Lu, Yuan Wu, Jiejie Dai

**Affiliations:** a Center of Tree Shrew Germplasm Resources, Institute of Medical Biology, Chinese Academy of Medical Sciences and Peking Union Medical College, Yunnan Key Laboratory of Vaccine Research and Development on Severe Infectious Diseases, Yunnan Innovation Team of Standardization and Application Research in Tree Shrew, Kunming, China; b Department of Acute Infectious Diseases Control and Prevention, Yunnan Provincial Centre for Disease Control and Prevention, Kunming, China; c State Key Laboratory of Infectious Disease Prevention and Control, Collaborative Innovation Center for Diagnosis and Treatment of Infectious Diseases, National Institute for Communicable Disease Control and Prevention, Chinese Center for Disease Control and Prevention, Beijing, China

**Keywords:** *Clostridioides difficile*, new ribotype, RT078, pathogenic ability, animal models

## Abstract

*Clostridioides difficile* is the predominant antibiotic-associated enteropathogen associated with diarrhoea or pseudomembranous colitis in patients worldwide. Previously, we identified *C. difficile* RT078 isolates (CD21062) from elderly patients in China, including two new ribotype strains (CD10010 and CD12038) belonging to the ST11 group, and their genomic features were also investigated. This study compared sporulation, spore germination, toxin expression, flagellar characteristics, and adhesion among these strains in vitro and analysed their pathogenic ability in vivo using animal models. The results showed sporulation and spore germination did not significantly differ among the three *C. difficile* strains. CD10010 and CD12038 showed higher transcriptional levels of toxins until 48 h; thereafter, the transcriptional levels of toxins remained constant among RT078, CD10010, and CD12038. RT078 showed a loss of flagellum and its related genes, whereas CD12038 showed the highest motility in vitro. Both CD10010 and CD12038 initially showed *flg* phase OFF, and the flagellar switch reversed to phase ON after 48 h in swim agar. Flagellar proteins and toxins were both upregulated when *flg* phase OFF changed to *flg* phase ON status, enhancing their pathogenic ability. CD12038 showed the highest adhesion to Hep-2 cells. Histopathology and inflammation scores demonstrated that CD12038 caused the most severe tissue damage and infection in vivo. The new ribotype strains, particularly CD12038, exhibit higher pathogenic ability than the typical RT078 strain, both in vitro and in vivo. Therefore, more attention should be paid to this new *C. difficile* strain in epidemiological research; further studies are warranted.

## Introduction

Currently, *Clostridioides difficile* is the predominant antibiotic-associated enteropathogen, which causes diarrhoea or even pseudomembranous colitis, in hospitalized patients worldwide [1,2]. The pathogenic mechanisms of *C. difficile* are often associated with its toxins, spores, flagellum, adhesion, and imbalance of the host gut microbiota [3,4]. Recent study showed the presence of a flagellar switch that controlled the phase-variable production of flagellar proteins and toxins in *C. difficile* [5]. In addition, our previous research revealed that tree shrew could be used as a novel animal model for *C. difficile* infection [6].

A recent study revealed that *C. difficile* could be divided into six clades, including clade 1–5 and clade C-I [7]. Among these, clade 2 and 5 were both important sources for human *C. difficile* infection (CDI) at present. The sequence type (ST) 1 belonged to clade 2, and the hyper-virulent PCR ribotype (RT) 027 isolates from clade 2/ST1 have already caused an epidemic and a global outbreak [8]. Besides, the ST 11 group of *C. difficile* belonged to clade 5, is another important infectious source in animal populations and is also responsible for a large part of CDIs in humans [9]. Genetic and genomic research on *C. difficile* in human and animal populations indicates that CDI is a zoonosis [10].

The ST11 group is a heterogeneous lineage of *C. difficile* and is usually associated with some PCR RT strains, including RT033, 045, 066, 078, 126, and 193. The most important ST11 strain lineage, the RT078 isolate, is a major reservoir or source of infection in animals and is also responsible for human infections in developed countries [11]. In fact, RT078 was the third isolated *C. difficile* RT for human CDI in Europe [12]. Previous research on genome and evolution of the RT078 and ST11 RT sub-lineages revealed that the ST11 group strains have a massive pan-genome and many important antimicrobial resistance elements, which contribute to the global transmission of this lineage [9].

Previously, during a systemic study of *C. difficile*, we identified six RT078 isolates, including two new RT strains, from elderly patients in China [13]. The two novel strains were not classified in any previously described types, and shared the different ribotypes. The genomic features of these isolates were investigated and some differences were found among CD21062 (RT078), CD10010 and CD12038 (new RT strains) [14]. However, the phenotype of these strains remains unknown, and functional studies on their pathogenic ability remain limited; knowledge regarding the existence of bacterial populations more virulent than the typical strains may be useful for future epidemiological research. In the present study, we performed a functional study on RT078, CD12038, and CD10010 and compared their pathogenic ability both in vitro and in vivo.

## Materials and methods

### Bacterial sources

CD21062 (RT078) and CD10010 and CD12038 (new RTs) strains were all isolated from elderly patients in a tertiary hospital in Beijing, China, and have been previously described [13]. All these *C. difficile* strains belong to the ST11 group and contain the toxin genes *tcdA*, *tcdB*, *cdtA*, and *cdtB*. The genomes of the strains have been deposited in the NCBI database with accession number PRJNA497978. The strains were cultured on brain heart infusion agar containing 10% cysteine (BHIS). Tryptose-yeast (TY) medium containing 3% tryptose, 2% yeast extract, and 0.1% thioglycolate was used for liquid culture. An anaerobic environment was maintained at all times using an anaerobic bag (MITSUBISHI, Japan). An incubation temperature of 37 °C was used for *C. difficile* culture.

### Sporulation and spore germination

The three strains were inoculated on BHIS agar medium under anaerobic conditions to induce sporulation. From day 4 to 7, on each day, the top of the agar medium was gently swiped with the inoculating loop and a small amount of culture was collected in a 1.5 ml microcentrifuge tube. These cultures were resuspended in 50 µl sterile water and the progress of sporulation was examined using light microscopy using a spore staining method (Wirtz-Conklin) [15]. Briefly, the spore suspension was smeared on a glass slide and fixed with flame, and then flooded with 5% malachite green (Huaikai, China). Slides were intermittently heated with a flame for 30 s, and rinsed with distilled water, finally counterstained with 0.5% Safranin-O (Huaikai, China) for 3 s. The spore preparation was accorded to previous method [16]. Strains were incubated under anaerobic conditions for seven days. All surface growth was extracted and transferred to microcentrifuge tubes containing 1 mL of sterile ice-cold water. The mixtures were centrifuged five times for 1 min at 13,000 × *g*. The washed pellets were suspended in 833 μL of 20% HistoDenz (Sigma-Aldrich) and combined into a 5 mL mixture. The suspension was gently layered onto 10 mL of 50% HistoDenz in a 15 mL centrifuge tube and centrifuged for 15 min at 15,000 × *g* at 4 °C. The spores were resuspended in 1 mL of sterile ice-cold water, and centrifuged for 1 min at 13,000 × *g*. The procedure was repeated five times to remove HistoDenz, and finally, the spore pellet of each strain was resuspended in 200 µl water and stored at 4°C [16].

To recover *C. difﬁcile* spores, BHIS agar containing 0.1% taurocholate was used. Each puriﬁed spore suspension was adjusted to 0.5 McF and serially diluted 10-fold; plates were inoculated with 100 µl of diluted spore suspension per plate [16]. After 48 h of anaerobic cultivation, the spore germination of the three strains was analysed by plate counting method.

### Quantitative reverse transcription PCR

Strains were cultured in TY medium at 12, 24, 48, and 72 h by inoculated a single colony of bacteria respectively. Total RNA of bacteria was extracted by using TRIzol reagent (Ambion). The relative expression of toxin genes and regulatory genes of *C. difficile* (*tcdA*, *tcdB*, *tcdC*, *tcdR*, *tcdE*, *cdtA* and *cdtB*) were analysed using the One-Step SYBR Green qRT-PCR kit (abm, CANADA) according to the manufacturer’s instructions. In brief, reactions were performed in a total volume of 20 µl, including 10 µl SYBR Green PCR master mix, 0.4 µl qRT-PCR enzyme mix, and forward and reverse primers 1 µl each, using a CFX-96 real-time PCR instrument (Bio-Rad). cDNA synthesis was performed at 42 °C for 15 min, and the amplification procedure was performed under the following conditions: 95 °C for 10 min, followed by 40 cycles of 95 °C for 15 s and 60 °C for 60 s. The data were normalized to *rpoA* gene expression of *C. difficile*, and three biological replicates were performed in triplicate. The primers used in quantitative reverse transcription PCR (qRT-PCR) based on previous studies [17,18] were listed in Table S1.

### ELISA

The three strains cultured in TY medium for 12, 24, 48, and 72 h were centrifuged to collect the supernatants containing TcdA and TcdB toxins; the supernatant was filtered with a 0.45 µm filter. Next, a 96-well plate was coated with the supernatant at 4 °C overnight and then washed with PBST (phosphate-buffered saline pH 7.4 + 0.05% Tween 20). The plate was blocked with 5% skim milk at 37 °C for 2 h. The polyclonal antibodies of TcdA and TcdB (chicken IgY; 1:1000 dilution; List Biological laboratories) were used for first hybridization; then, goat anti-chicken IgY secondary antibody, (HRP-conjugated; 1:5000 dilution; Invitrogen) was used. The TMB chromogenic reagent kit (Sangon, China) was used to determine the absorbance at 450 nm. Pure toxin A and toxin B (List Biological laboratories) were used as positive controls, and the standard curves were obtained using 20, 2, 0.2, 0.02, and 0.002 ng/ml of TcdA and TcdB [19].

### Cytotoxicity assays

Monolayer Vero cells were prepared by seeding 96-well plates with 100 µl of cell suspension (10^5^ cells/ml) cultured with DMEM with glutamine supplemented with 10% foetal bovine serum at 37 °C containing 5% CO_2_ for 24 h. Each supernatant obtained from *C. difficile* cultures (described above) was subjected to four-fold serial dilutions, and 20 µl of each dilution was added to the cells. After 24 h incubation, the cells were examined by light microscopy (Nikon) to determine the toxin endpoint titre for each supernatant. The endpoint titres were expressed as the highest dilution showing 50% cytopathic effect (CPE) [20].

### Motility assays

Swarming and swimming motility of *C. difficile* was analysed as previously described [21]. Brain heart infusion (BHI) broth medium containing 0.3% and 0.4% agar constituted swim and swarm agar, respectively. Swim agar plates were stab inoculated with each strain at the same cell density and then incubated at 37 °C for 48 h. Swarm agar plates were spot inoculated with each strain and incubated as described above. Both experiments were quantitatively determined by measuring the radius or zones. Motility assays were performed in six replicates for each strain and repeated independently three times.

Specifically, swimming motility assays were performed 24, 48, 72, and 96 h after inoculation to determine the flagellar switch of the three *C. difficile* strains. Electron microscopy was used to examine the presence of flagellar structures on the surface of *C. difficile* strains as described previously [22]. Orientation-specific PCR was used to detect the *ﬂg* ON and OFF states during the experimental process [5]; *tcdA* and *tcdB* gene expression was analysed by qRT-PCR in the *ﬂg* ON and OFF states (primers were listed in Table S1), and western blotting was used to detect the TcdA and TcdB protein expression in the three strains in the *ﬂg* ON and OFF states. In brief, bacterial lysate proteins were separated using 8% SDS-PAGE and transferred to PVDF membranes (Bio-Rad). TcdA and TcdB were detected using anti-TcdA and anti-TcdB antibodies (chicken IgY; 1:1000 dilution; List Biological laboratories) and goat anti-chicken IgY secondary antibody (HRP-conjugated; 1:2000 dilution; Invitrogen) was used. The images were visualized using the ChemiDoc MP imaging system (Bio-Rad).

### Adherence to cells

*Clostridioides difficile* strains grown to log phase were used, the concentration of bacteria were adjusted to 0.5 McF (5 × 10^5^ CFU/ml), and then 1 ml suspension was added to Hep-2 cells in 6-well plates respectively. Each well contained 2 × 10^6^ Hep-2 cells. The plates were incubated for 2 h anaerobically, and then washed with PBS to remove the non-adherent bacterial cells. The adherent cells were scraped from the wells, and serially diluted and cultured on BHIS agar plates. Percentage adherence was calculated as follows: count of bacteria recovered/count of bacteria added × 100%. The cells were examined by light microscopy (Nikon), and three biological replicates were performed in triplicate reactions.

### Animal experiments

Tree shrews and BALB/c mice were both used for animal experiments, respectively. The laboratory procedure using tree shrew was based on our previous study [6]. Tree shrews were from a closed population, and were healthy animals without visible signs of tumours or disease. Ten of the animals were male and 10 were female. Tree shrews were housed in 20 sterile cages containing autoclaved water and irradiated food. We divided these animals into four groups that were infected with the CD21062, CD10010, or CD12038 strain of *C. difficile* and one control group without any administration. Each group contained five animals. The experimental animals were received an antibiotic pre-treatment [23]. In general, tree shrews received the antibiotic cocktail for 7 days in the drinking water [metronidazole (0.215 mg/mL), vancomycin (0.045 mg/mL), kanamycin (0.4 mg/mL), gentamicin (0.035 mg/mL) and colistin (850 U/mL)]. A single intraperitoneal injection of clindamycin (10 mg/kg) was administered one day prior to infection. Tree shrews were then infected by oral gavage of 10^5^ spores of *C. difficile*. The animals were observed daily for signs of disease (i.e. diarrhoea or ruffled fur), and seven days after gavage, the tree shrews were euthanized by intraperitoneal injection of 2% pentobarbital sodium (0.2 ml/100 g, Sigma-Aldrich, USA). Histopathological analysis and scoring were performed blindly by a pathologist for ileal and colonic tissues of tree shrews. Briefly, the inflammation score parameters contained lymphocyte infiltration, elongation or distortion of crypts, thickening of the bowel wall and frank ulceration [24]. The degree of inflammation of tissues were graded semi-quantitatively from 0 to 4, which indicated that 0 was no inflammation found; 1 was low lymphocyte infiltration with infiltration seen below 10%, and no structural changes; 2 was moderate lymphocyte infiltration with infiltration from 10% to 25%, bowel wall thickening and crypt elongation, but no ulceration found; 3 was high degree of lymphocyte infiltration with infiltration from 25% to 50%, thickening of bowel wall extended beyond mucosal layer and high vascular density; 4 was highest lymphocyte infiltration with infiltration above 50%, transmural bowel wall thickening with ulceration and crypt elongation with distortion.

Gut microbiota changes during the experiment were assessed to determine the characteristics of the infections with the three strains. Faecal samples were collected at baseline, during antibiotic treatment, and after *C. difficile* infection. Briefly, faecal samples were collected before antibiotics usage; faeces were collected after antibiotics feeding at day 6; day 5 of *C. difficile* infection in tree shrew was collected for the third time. Total genomic DNA was extracted using a faecal DNA extraction kit (Tiangen, Beijing) according to the manufacturer’s instructions [25]. The V3-V4 variable region of the 16S rRNA gene was amplified using previously described primers [26]. Library preparation followed guidelines from Illumina (Illumina, USA). Bioinformatics analysis was performed as described previously [27], and sequence data were deposited in the NCBI database (SRA accession: PRJNA541587).

Toxin levels of the infected tree shrew stools were tested by ELISA method. After *C. difficile* infection, the fresh faecal samples of tree shrews were collected for a week, and suspended to 0.5 g/ml concentration by PBS buffer (pH 7.4), then coated in 96-well plate at 4 °C overnight. The experimental procedure was the same as in vitro ELISA method mentioned above.

Mice experiment was used to evaluate the biological replicability and reliability results of tree shrews. The identical laboratory procedure for antibiotic pre-treatment, infected dose and histopathological examination were performed as mentioned above.

The animal experiments were carried out in accordance with relevant guidelines and regulations and were approved by the Ethics Committee of the Institute of Medical Biology, Chinese Academy of Medical Sciences and Peking Union Medical College.

### Statistical analysis

Statistical analyses were performed using SPSS (version 16.0, IBM, USA). The Kolmogorov-Smirnov test, t-test, ANOVA, or Kruskal-Wallis H test was used as appropriate. A P value <0.05 was considered to indicate statistical significance.

## Results

### Sporulation and spore germination

**Figure 1.**
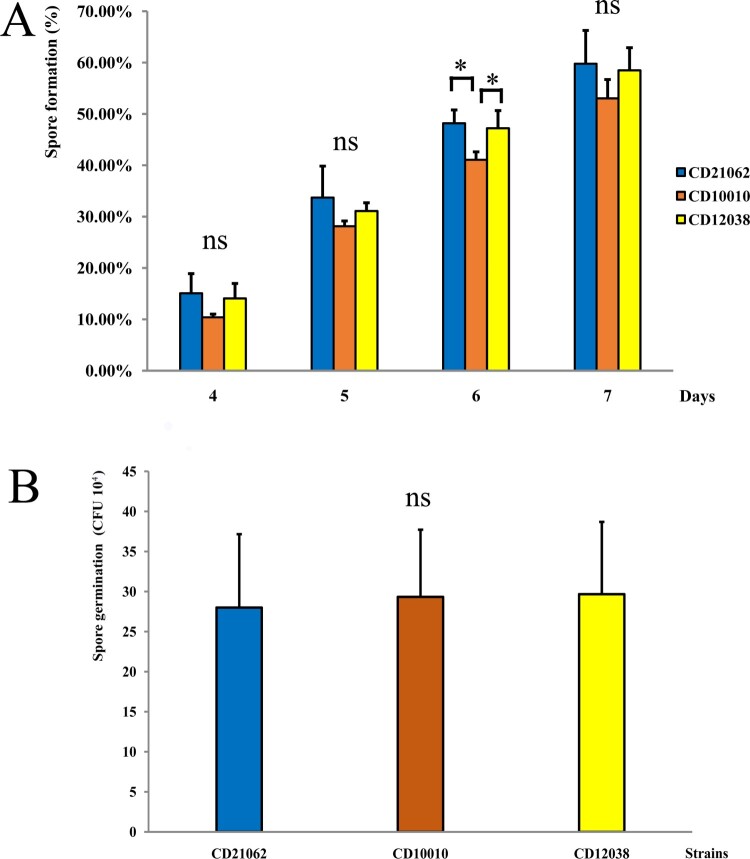
The sporulation and spore germination results of three *C. difficile* strains in this study. (A) The spore formation results of three strains from day 4 to 7. The blue colour represented CD21062; orange colour represented CD10010; yellow colour represented CD12038. The asterisk indicated statistical significance, and “ns” indicated not statistically significant for the pairwise comparisons. (B) The spore germination results of three *C. difficile* strains at 48 h. The blue colour represented CD21062; orange colour represented CD10010; yellow colour represented CD12038. The “ns” indicated not statistically significant for the pairwise comparisons.

The spore staining experiment started from day 4 of the bacterial culture, and the results showed no statistical difference in the spore formation rates of the three strains from day 4 to 7, except at day 6 (Figure 1(A)). The sporulation rate of CD10010 was lower than that of CD21062 and CD12038 on day 6. The sporulation rate of the three strains gradually increased with the culture time, and the average rate was approximately 60% on day 7. Spore germination did not differ significantly among the three *C. difficile* strains after 48 h of anaerobic culture. The bacterial count of CD21062, CD10010, and CD12038 was 28 ± 9.17 × 10^4^ CFU, 29.33 ± 8.39 × 10^4^ CFU, and 29.67 ± 9.02 × 10^4^ CFU, respectively (*F* = 0.030, *P* = 0.971) (Figure 1(B)). The spore staining of three *C. difficile* strains and spore germination results were shown in Figure S1A–F.

### Toxin expression and cytotoxicity

**Figure 2.**
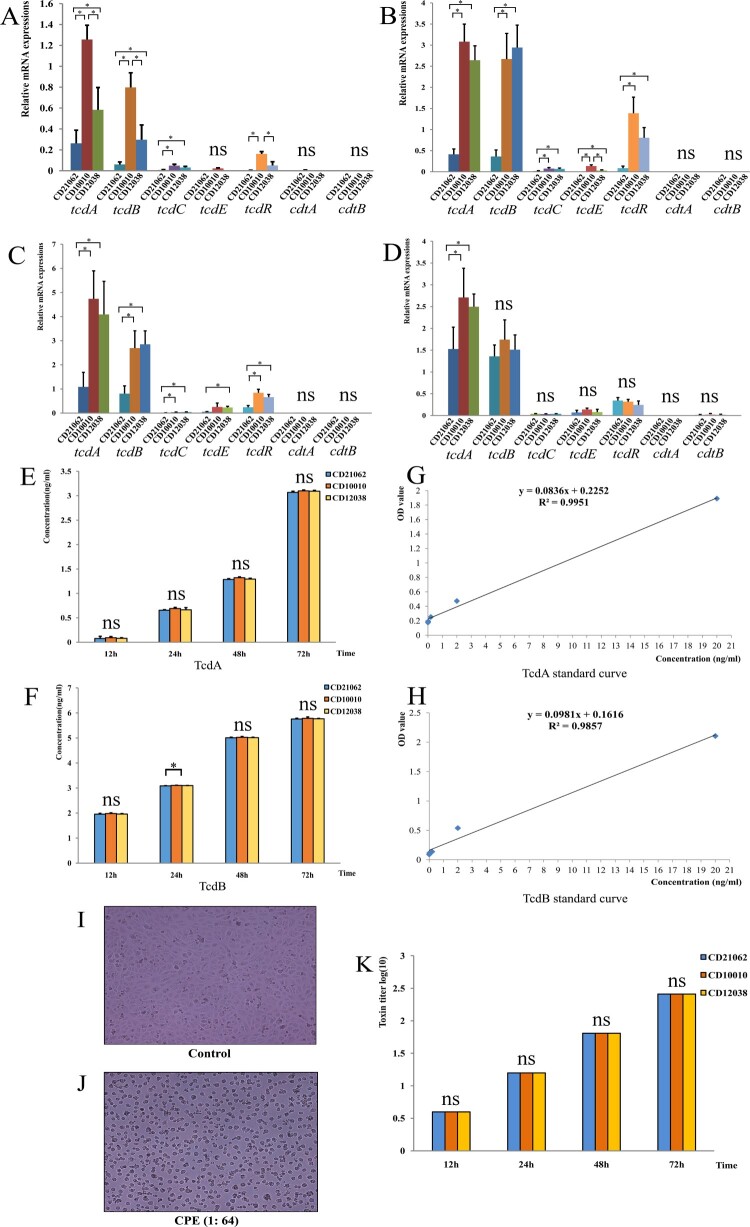
Toxin expression and cytotoxicity assays in three ST11 strains (CD21062, CD10010, and CD12038) of *C. difficile*. (A) Relative expression of toxin and regulatory genes at 12 h. The relative expressions of each gene among three strains were represented by different colours. The asterisk indicated statistical significance and “ns” indicated not statistically significant for the pairwise comparisons. (B) Relative expression of toxin and regulatory genes at 24 h. The relative expressions of each gene among three strains were represented by different colours. The asterisk indicated statistical significance and “ns” indicated not statistically significant for the pairwise comparisons. (C) Relative expression of toxin and regulatory genes at 48 h. The relative expressions of each gene among three strains were represented by different colours. The asterisk indicated statistical significance and “ns” indicated not statistically significant for the pairwise comparisons. (D) Relative expression of toxin and regulatory genes at 72 h. The relative expressions of each gene among three strains were represented by different colours. The asterisk indicated statistical significance and “ns” indicated not statistically significant for the pairwise comparisons. (E) TcdA quantification at different time points in the three strains. The blue colour represented CD21062; orange colour represented CD10010; yellow colour represented CD12038 and “ns” indicated not statistically significant for the pairwise comparisons. (F) TcdB quantification at different time points in the three strains. The blue colour represented CD21062; orange colour represented CD10010; yellow colour represented CD12038. The asterisk indicated statistical significance and “ns” indicated not statistically significant for the pairwise comparisons. (G) TcdA standard curve obtained using ELISA. (H) TcdB standard curve obtained using ELISA. (I) Normal (control) Vero cells visualized with light microscope (200×). (J) Typical cytopathic effects on Vero cells infected with *C. difficile* toxins (200×). (K) Toxin titre for cytopathic effects among three strains at different time points. The blue colour represented CD21062; orange colour represented CD10010; yellow colour represented CD12038. The “ns” indicated not statistically significant for the pairwise comparisons.

Study of the relative expression of toxin and regulatory genes of *C. difficile* revealed that CD21062 had lower expression levels of *tcdA*, *tcdB*, *tcdC*, *tcdE*, and *tcdR* than CD10010 and CD12038 until 48 h. In general, *tcdA* relative expression of CD10010 was 4.85 fold changes than CD21062 at 12 h, 7.33 at 24 h, 4.31 at 48 h, and 1.77 fold changes at 72 h. The *tcdA* relative expression of CD12038 was 2.23 fold changes than CD21062 at 12 h, 6.29 at 24 h, 3.75 at 48 h, and 1.64 at 72 h. The *tcdB* expression of CD10010 was 13.2 fold changes than CD21062 at 12 h, 7.22 at 24 h, and 3.21 at 48 h. The *tcdB* relative expression of CD12038 was 4.83 fold changes than CD21062 at 12 h, 8.17 at 24 h, and 3.52 at 48 h. A difference in the expression levels of *tcdA* and *tcdB* genes between CD10010 and CD12038 was found only at 12 h; thereafter, there was no statistically significant difference in the expression of these genes. At 72 h, the transcriptional levels of virulent and regulatory genes tended to be consistent among the three strains, and only a statistically significant difference was observed only for *tcdA* gene expression between CD21062 and CD10010, as shown in Figure 2(A–D).

The protein expression levels of TcdA and TcdB at 12, 24, 48, and 72 h were not significantly different among the three strains; however, CD10010 showed a higher TcdB level than CD21062 at 24 h (Figure 2(E,F)). Although the TcdA and TcdB protein levels were slightly higher in CD10010 than in the other strains, the difference was not statistically significant. The standard curve of TcdA and TcdB proteins was shown in Figure 2(G,H).

The cytotoxicity assays were performed with bacterial culture supernatants, and the CPE was observed with the serial dilutions of cultures using a light microscope, as shown in Figure 2(I,J). The results showed that the CPE produced by dilutions of the three *C. difficile* strains were consistent at 12, 24, 48, and 72 h. At 48 h, CPE was observed at the 1:64 dilution, whereas at 72 h, CPE was observed at the 1:256 dilution (Figure 2(K)).

### Motility assay

**Figure 3.**
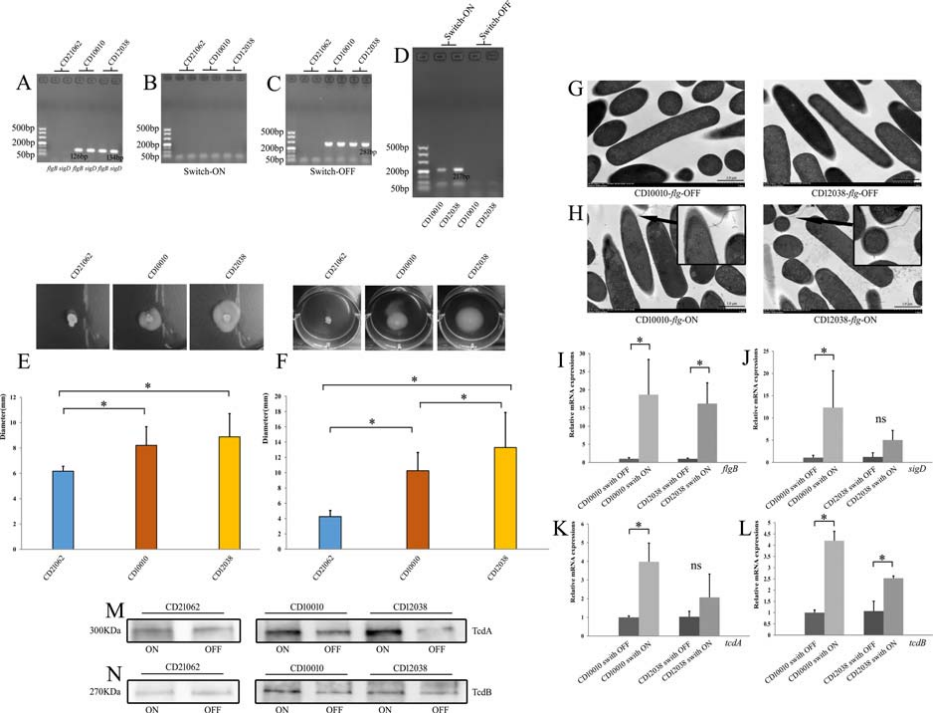
Motility assays and flagellar switch-related assays in RT078 and two new ribotype strains of *C. difficile*. (A) PCR results for early flagellar biosynthesis operon genes. (B) Orientation PCR results for flagellar switch ON in three strains cultured in BHI agar. (C) Orientation PCR results for flagellar switch OFF in three strains cultured in BHI agar. (D) Orientation PCR results for when *flg* phase OFF changed to *flg* phase ON in CD10010 and CD12038. (E) Swarming motility assay for CD21062, CD10010, and CD12038. The blue colour represented CD21062; orange colour represented CD10010; yellow colour represented CD12038. The asterisk indicated statistical significance. (F) Swimming motility assay for CD21062, CD10010, and CD12038. The blue colour represented CD21062; orange colour represented CD10010; yellow colour represented CD12038. The asterisk indicated statistical significance. (G) Transmission electron microscopy of two ribotype strains at *flg* phase OFF status. (H) Transmission electron microscopy of two ribotype strains at *flg* phase ON status (the arrows indicate flagella). (I) Relative *flgB* expression for *flg* phase OFF and ON status for two new ribotype strains. The asterisk indicated statistical significance. (J) Relative *sigD* expressions for *flg* phase OFF and ON status for two new ribotype strains. The asterisk indicated statistical significance and “ns” indicated not statistically significant for the pairwise comparisons. (K) Relative *tcdA* expressions for *flg* phase OFF and ON status for two new ribotype strains. The asterisk indicated statistical significance and “ns” indicated not statistically significant for the pairwise comparisons. (L) Relative *tcdB* expressions for *flg* phase OFF and ON status for two new ribotype strains. The asterisk indicated statistical significance. (M) Western blot results of TcdA for *flg* phase OFF and ON status for CD21062, CD10010, and CD12038. (N) Western blot results of TcdB for *flg* phase OFF and ON status for CD21062, CD10010, and CD12038.

In silico analysis based on the complete genomes of these three *C. difficile* strains indicated that CD21062 (accession number: CP033216) showed a loss of most flagella-related genes, such as *flgG*, *fliM*, *fliN*, and flagellar switch genes (Figure S2). However, the new RTs showed an inverse orientation of the flagellar switch (switch OFF). In addition, multiple sequence alignments indicated that the flagellar switch genes of CD10010 and CD12038 were similar to those of the BI1 strain in a 154 bp region owing to a DNA inversion between the inverted repeats (Figure S3). PCR also confirmed that CD21062 has lost *flgB* and *sigD*, the early flagellar biosynthesis operon downstream of the flagellar switch genes (Figure 3(A)). The new RT strains had OFF orientation of the flagellar switch when cultured in BHI agar, but the orientation of the flagellar switch was reversible during swimming because positive PCR results were obtained only in the ON state of the flagellar switch (Figure 3(B–D)).

Swarming motility of the three *C. difficile* strains differed significantly (*F* = 12.625, *P* = 0.000; Figure 3(E)). CD21062 did not exhibit swarming motility, and CD12038 showed the highest motility. Swimming motility also differed significantly (*F* = 27.781, *P* = 0.000) among these strains; CD12038 had the strongest motility in the medium at 48 h (Figure 3(F)). Furthermore, approximately 30% and 100% of CD10010 and CD12038 colonies showed swimming motility in the medium at 48 and 72 h, respectively, but no swimming phenomenon was observed for CD21062 at any time point. Transmission electron microscopy further verified that CD10010 and CD12038 cells had no flagella in *flg* OFF state (Figure 3(G)), whereas flagella could be visualized in *flg* ON state (Figure 3(H)) for both new RTs of *C. difficile*.

Because the orientation of the flagellar switch controls the expression of downstream flagellar genes and toxin genes, we further analysed the gene expression in the two new RT strains in *flg* OFF and ON states. qRT-PCR showed that the downstream flagellar genes *flgB* and *sigD* were both upregulated when the flagellar switch orientation OFF reversed to ON (Figure 3(I,J)). The expression of *tcdA* and *tcdB* was also upregulated when the flagellar switch underwent DNA inversion (Figure 3(K,L)); moreover, western blot indicated that both TcdA and TcdB toxins were upregulated in accordance with the gene expressions (Figure 3(M,N)).

### Adherence to cells

**Figure 4.**
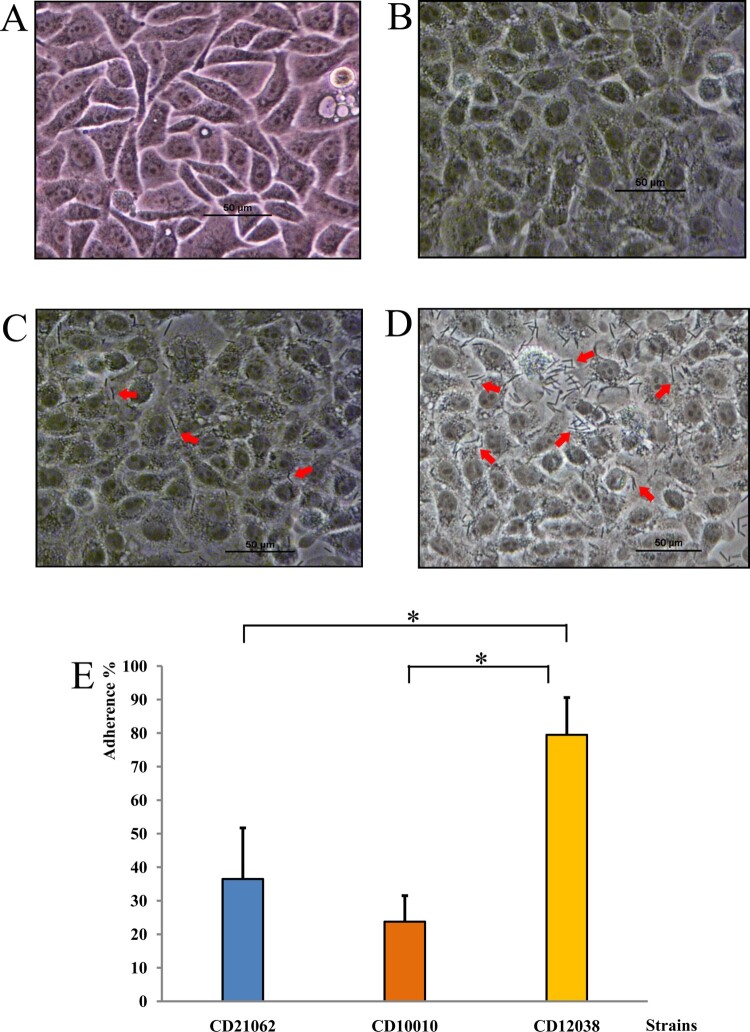
Adherence to Hep-2 cells of three *C. difficile* strains. (A) Normal Hep-2 cells (200×). (B) CD21062-infected Hep-2 cells (200×). (C) CD10010-infected Hep-2 cells (200×; the arrows indicate bacteria adhered to Hep-2 cells). (D) CD12038-infected Hep-2 cells (200×; the arrows indicate bacteria adhered to Hep-2 cells). (E) Adhesion ability of the three strains. The blue colour represented CD21062; orange colour represented CD10010; yellow colour represented CD12038. The asterisk indicated statistical significance.

Microscopic observation indicated that the ability of *C. difficile* to adhere to Hep-2 cells differed among the three strains; CD12038 displayed the highest adhesion ability. A higher number of bacteria could be observed adhered to Hep-2 cells in CD12038 than in CD21062 and CD10010 (Figure 4(A–D)). In addition, the adhesion rate of CD12038 (79.49 ± 11.13%) was significantly higher (*F* = 26.395, *P* = 0.000) than that of CD21062 (36.48 ± 15.25%) and CD10010 (23.77 ± 7.75%), as Figure 4(E) shown.

### Animal experiments

**Figure 5.**
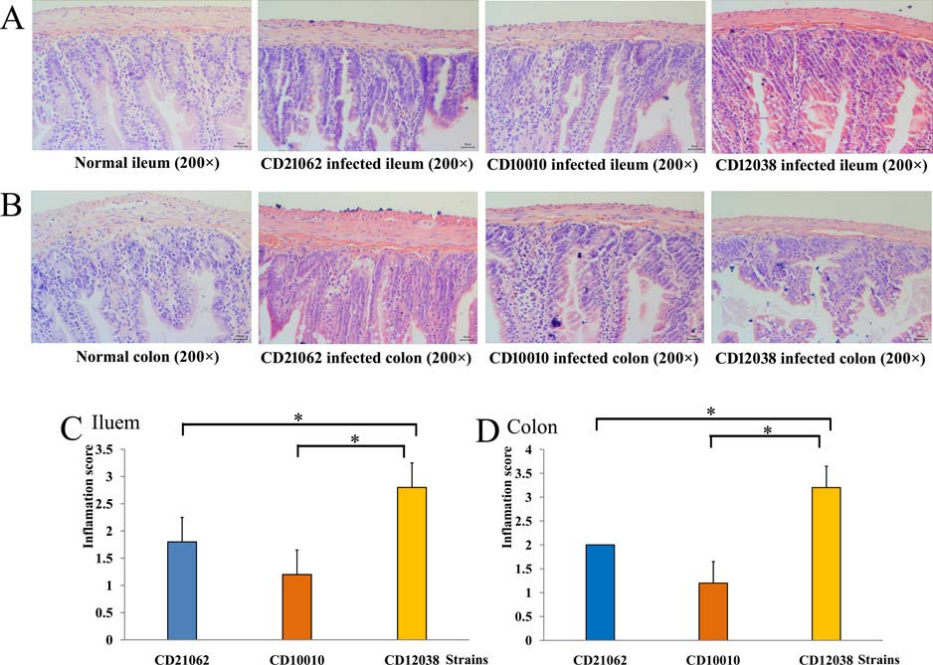
Histopathological examinations of tree shrews infected with three *C. difficile* strains. (A) Histopathological examination of the ileum in animals infected with *C. difficile.* (B) Histopathological examination of the colon in animals infected with *C. difficile.* (C) Inflammation scores of the ileum in animals infected with three strains. The blue colour represented CD21062; orange colour represented CD10010; yellow colour represented CD12038. The asterisk indicated statistical significance. (D) Inflammation scores of the colon in animals infected with three strains. The blue colour represented CD21062; orange colour represented CD10010; yellow colour represented CD12038. The asterisk indicated statistical significance.

In vivo analysis by using tree shrews indicated that *C. difficile* colonized the gastrointestinal tract of the tree shrews and caused diarrhoea and weight loss [6]. In the CD21062 infection group, the ileal epithelial cells were destroyed, and structural disorder, hyperaemia, oedema, inflammatory cell infiltration were observed; in the CD10010 infection group, inflammatory cell infiltration was observed in the mucosal and submucosal layers with crypt destruction; in the CD12038 infection group, ileal epithelial cells were destroyed, and structural disorder, mucosal ulcers, and necrosis could be observed; some lesion sites reached the mucosal muscle layer, and gland destruction, crypt destruction, goblet cell number reduction, and inflammatory cell infiltration in the mucosal and submucosal layers could be visualized (Figure 5(A)). The colon tissue of the CD21062 infection group showed increased eosinophils and lymphocyte and plasma cell infiltration in the mucosal, submucosal, and muscle layers; in the CD10010 infection group, the colon tissue showed structural destruction and inflammatory cell infiltration; in the CD12038 infection group, the colonic mucosa and submucosa showed structural destruction, and the mucosal, submucosal, and muscle layers showed increased eosinophils with lymphocyte and plasma cell infiltration (Figure 5(B)). The inflammation scores showed that infection with CD12038 caused the highest degree of inflammation both in the ileal and colonic tissues, followed by that with CD21062 and CD10010, respectively (*F* = 16.333, *P* = 0.000 for ileum; *F* = 38.000, *P* = 0.000 for colon; Figure 5(C,D)).

The gut microbiota changes grouped by three *C. difficile* strains revealed that CD12038 showed the highest number of sequencing reads for genus *Clostridium* among these strains (Figure S4A), indicating that it had the strongest growth and replication functions in the gastrointestinal tract of tree shrews. Gut microbiota taxa grouped by the three strains indicated that CD12038 had a relatively higher abundance of *Clostridium* than CD21062 and CD10010, but the difference was not statistically significant (Figure S4B and C). To eliminate the influence of the baseline gut microbial community on this experiment, we further compared the OTUs grouped by three strains and each animal during the experimental process. The results indicated a higher relative abundance of *Clostridium* at the genus level in CD12038-infected animals, and a higher abundance of *Clostridium* could be found in CD12038-infected tree shrews, especially for animal number 13 and 14 after infection, as Figure S5 shown. Because the *Clostridium* was eliminated after antibiotics treatment in tree shrews, the abundance of genus *Clostridium* correctly reflected the growth and replications of strains after *C. difficile* infection. The total sequencing reads counts of genus *Clostridium* after infection was 76,912 reads for CD12038, 15,546 reads for CD21062, and 21,288 reads for CD10010.

**Figure 6.**
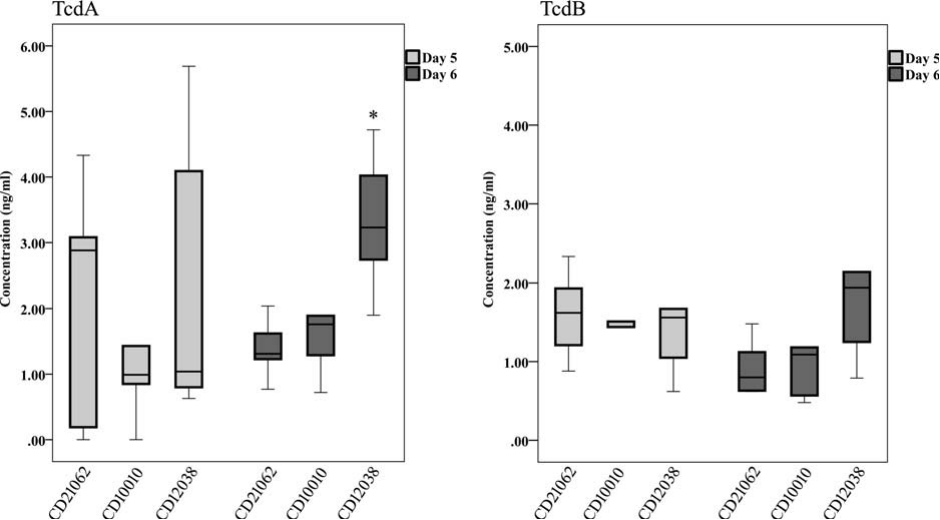
The TcdA and TcdB toxins levels of infected tree shrew faeces in this study. TcdA toxin levels were shown on the left at day 5 and 6 after infection. The asterisk indicated statistical significance. TcdB toxin levels were shown on the right at day 5 and 6 after infection.

At experimental day 5, most of the tree shrews faeces could be detected the toxins (Figure 6). The TcdA concentration of CD21062 at day 5 was 2.10 ±1.91 ng/ml, 1.42±1.44 ng/ml for CD10010, and 2.45±2.30 for CD12038. Statistical significance of TcdA was only found at day 6; CD12038 showed the highest level (3.32±1.10 ng/ml). For the TcdB toxin levels in animal stools, the concentrations of CD21062, CD10010 and CD12038 at day 5 were 1.59±0.57 ng/ml, 1.40±0.39 ng/ml, 1.55±0.83 ng/ml respectively. At day 6, 0.93±0.37 ng/ml, 1.12±0.72 ng/ml, and 2.21±1.61 ng/ml of TcdB toxin levels were detected for CD21062, CD10010 and CD12038, no statistical significance was found.

**Figure 7.**
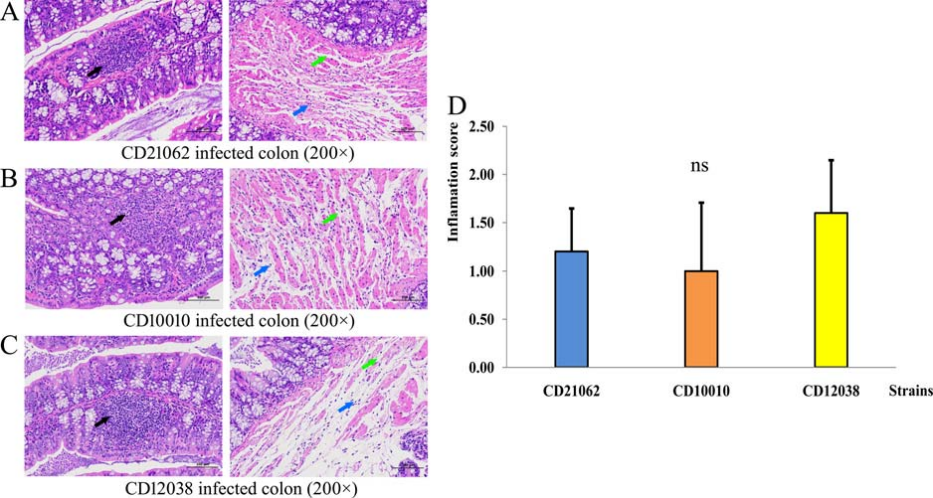
Histopathological examinations of BALB/c mice infected with three *C. difficile* strains. (A) Histopathological examination of the colon in mice infected with CD21062. The black arrow indicated lymphocyte infiltration; green arrow indicated loose cell arrangement, and widened cell spacing; blue arrow indicated a small amount of lymphocyte. (B) Histopathological examination of the colon in mice infected with CD10010. The black arrow indicated lymphocyte infiltration; green arrow indicated loose cell arrangement, and widened cell spacing; blue arrow indicated a small amount of lymphocyte. (C) Histopathological examination of the colon in mice infected with CD12038. The black arrow indicated lymphocyte infiltration; green arrow indicated loose cell arrangement, and widened cell spacing; blue arrow indicated a small amount of lymphocyte. (D) Inflammation scores of the colon in animals infected with three strains. The blue colour represented CD21062; orange colour represented CD10010; yellow colour represented CD12038.

No BALB/c mice died during the experimental process in this study. Histopathological results showed that the colonic glands of mice were abundant, with lots of lymphocyte infiltration (Figure 7(A–C), black arrow). The local structure of the colonic muscle layer was destroyed, with loose cell arrangement, and widened cell spacing (green arrow in Figure 7(A–C)), and a small amount of lymphocyte infiltration could be identified (blue arrow in Figure 7(A–C)). This pathological change was similar between the three strains, but the inflammation score results showed that CD12038 was still higher than that of CD21062 and CD10010 (Figure 7(D)).

## Discussion

*Clostridioides difficile* RT078 is the primary ST11 sub-lineage, frequently found in livestock and retail meat products [11]. It was also the main infection source for both hospital-acquired and community-acquired CDIs in North America and Europe [28,29]. Previously, we identified three ST11 group *C. difficile* strains with different RTs from elderly patients, and the whole genome sequence of three ST11 group *C. difficile* strains have been reported before [13,14]. The results showed that these isolates had identical point mutations and deletions in the *tcdC* gene. CD21062 and CD12038 contained plasmids but CD10010 did not; the number of transposons they had and their genetic organization also differed, especially for bacterial CRISPR spacers. CD21062 carried different antibiotic resistance genes and showed a loss of many flagellum-related genes. However, most studies on the pathogenic mechanism or phenotype of *C. difficile* focus on RT027 strains, whereas few focus on RT078 isolates. Therefore, it was important to analyse the pathogenic ability and phenotype of RT078, including other newly found strains, both belonging to the ST11 group.

The major virulence toxins TcdA and TcdB are essential for CDIs in hospitals [30]. All the ST11 strains in this study had these virulence genes, and they could be considered as hyper-virulent strains. The two new RT *C. difficile* strains showed higher expression levels of toxin genes until 48 h of culture; thereafter, that the transcriptional levels of the toxin genes tended to be constant among RT078 and the two new RTs. The results also suggested that *tcdA* and *tcdB* are early transcription genes; The expression levels of these genes gradually increased between 1 and 24 h, were the highest at 48 h, and then gradually stabilized.

Flagella are known to be involved in the pathogenic process of CDIs, but their role remains unclear and varies in different strains. A previous study showed that the genes governing the flagellar assembly in *C. difficile* had three operons [30]. The F3 locus contains early-stage genes such as sigma factor *SigD*, and *SigD* controls the expression of late-stage flagellar genes at the F1 locus, such as *fliC* and *fliD.* The F2 locus is responsible for post-translational modifications of flagella [31,32]. Remarkably, the F3 locus was lost in the virulent 078 lineage *C. difficile*, suggesting that flagella and motility are dispensable in this strain. The present study also demonstrates that the RT078 strain has lost the early flagellar biosynthesis operon and flagellar switch, and the RT078 strain showed no migration ability. However, the two new RT strains possessed flagellum-related operons, and swimming motility was observed in the medium after 48 h culture. Previously, Anjuwon-Foster et al. reported the presence of a flagellar switch that controls the phase-variable production of flagellar proteins and toxins in *C. difficile* [5,33]. In their study, RT012, RT017, and RT027 strains were used, and they found bacteria in *flg* phase ON state expressed flagellar proteins and toxins, whereas their expression was attenuated in the inverse orientation, defined as *flg* phase OFF. They further evaluated multiple isolates of RT012 strains and found them to be primarily flagellar phase off. Some of the isolates showed the ability to switch between on and off status. They concluded that *C. difficile* RT012 strains showed heterogeneity in phase-variable production of flagellar proteins and toxins, which may occur in other pathogenic *C. difficile* strains [22]. For the ST11 group of pathogenic *C. difficile* isolates in this study, similar findings were obtained for heterogeneity in phase variation. RT078 did not exhibit phase variation of *C. difficile* virulence factors, but the two new RT strains initially showed *flg* phase OFF and then switched to phase ON after 48 h in swim agar. Flagellar proteins and toxins were upregulated when the *flg* phase OFF changed to the *flg* phase ON status, which enhanced the pathogenic ability of the isolates in vitro.

The tree shrew (*Tupaia belangeri*) belongs to the family Tupaiidae, widely distributed in South and Southeast Asia. Tree shrews are considered a more effective animal model than rodents as an alternative for non-human primates [34]. The disease symptoms, histopathology, and gut microbiota changes following a *C. difficile* infection in tree shrews are similar to those observed in humans, and we considered tree shrews to be a useful animal model for the study of CDIs. Histopathology and inflammation scores of intestinal tissues demonstrated that CD12038 provoked the most serious tissue damage and infection in vivo. The gut microbiota changes grouped by three strains also suggested that CD12038 had the strongest growth and replication functions in the gastrointestinal tract of tree shrews. In vivo experiments in mice confirmed the availability and effectiveness of tree shrews as a novel laboratory animal for CDI, and also confirmed the different pathogenic abilities among three *C. difficile* by using different animal models. The histopathological scores of the colon of mice were consistent with the results of intestinal inflammation of tree shrews, both of which indicated that CD12038 caused the highest degree of infection and lesion intensity in host animals. These findings confirm that the new RT strains, particularly CD12038, have a higher pathogenic ability than typical RT078 strains both in vitro and in vivo, both belonging to the ST11 group of *C. difficile*. Future epidemiological research should focus more attention on this emerging *C. difficile* strain.

## Supplementary Material

Supplementary_Table_S1.doc

Supplementary_figure_S5.tif

Supplementary_figure_S4.tif

Supplementary_figure_S3.tif

Supplementary_figure_S2.tif

Supplementary_figure_S1.tif
