# Supplementary material for: New ribotype Clostridioides difficile from ST11 group revealed higher pathogenic ability than RT078
Source: Emerg Microbes Infect. 2021 Apr 5;10(1):687–99. doi: 10.1080/22221751.2021.1900748 (PMC8023612; doi:10.1080/22221751.2021.1900748)
Supplement: Supplementary_Table_S1.doc [file TEMI_A_1900748_SM1179.doc]

Table S1. Primers used in this study

| **Primers** | **Sequence (5'-3')** | **Use** |
| --- | --- | --- |
| tcdA-F1 | GTCGGATTGCAAGTAATTGACAATA | qRT-PCR |
| tcdA-R1 | TAACAGTCTGCCAACCTTTTGAGA | qRT-PCR |
| tcdB-F1 | ACCATATAGCTTTGTAGATAGTGAAGGAAA | qRT-PCR |
| tcdB-R1 | AAGAACTACATCAGGTAATTCAGATACAAA | qRT-PCR |
| tcdC-F1 | AGGGTATTGCTCTACTGGCATTTATT | qRT-PCR |
| tcdC-R1 | CCTCATGGTCTTCAGAACAAGCT | qRT-PCR |
| tcdR-F1 | CAAGAAATAACTCAGTAGATGATTTGCAA | qRT-PCR |
| tcdR-R1 | TCTCCCTCTTCATAATGTAAAACTCTACTAAG | qRT-PCR |
| tcdE-F1 | AACATCTTGGAATATCTGAATTTTTCTCTA | qRT-PCR |
| tcdE-R1 | TCTGTCATTGCATCTAGTAAAATTGCT | qRT-PCR |
| rpoA-F1 | GGATGATATGATGAAGGTTAGAAACCT | qRT-PCR |
| rpoA-R1 | CCCAATCCAAGTTCTTCTAGTTTTTG | qRT-PCR |
| cdtA-F1 | GGGAAGCACTATATTAAAGCAGAAGC | qRT-PCR |
| cdtA-R1 | CTGGGTTAGGATTATTTACTGGACCA | qRT-PCR |
| cdtB-F1 | TTGACCCAAAGTTGATGTCTGATTG | qRT-PCR |
| cdtB-R1 | CGGATCTCTTGCTTCAGTCTTTATAG | qRT-PCR |
| flgB-F | GCAACTAATCTAAGAAGTCAGACAATAGC | PCR and qRT-PCR |
| flgB-R | AGGCATAGCATCATTTAGTGTTTCTTC | PCR and qRT-PCR |
| sigD-F | GAATATGCCTCTTGTAAAGAGTATAGCA | PCR and qRT-PCR |
| sigD-R | TGCATCAATCAATCCAATGACTCC | PCR and qRT-PCR |
| qPCR_FlgSwit-ON | GTTTTCTTACCAAAGTGATACATTATTATATTAATG | qRT-PCR |
| qPCR_FlgSwit-OFF | CATTAATATAATAATGTATCACTTTGGTAAGAAAAC | qRT-PCR |
| qPCR_FlgSwit-REV | GCTATTGTCTGACTTCTTAAATTAGTTGCAT | qRT-PCR |
| rpoCqF | CTAGCTGCTCCTATGTCTCACATC | qRT-PCR |
| rpoCqR | CCAGTCTCTCCTGGATCAACTA | qRT-PCR |
