# Supplementary figures and images for: New ribotype Clostridioides difficile from ST11 group revealed higher pathogenic ability than RT078
Source: Emerg Microbes Infect. 2021 Apr 5;10(1):687–99. doi: 10.1080/22221751.2021.1900748 (PMC8023612; doi:10.1080/22221751.2021.1900748)

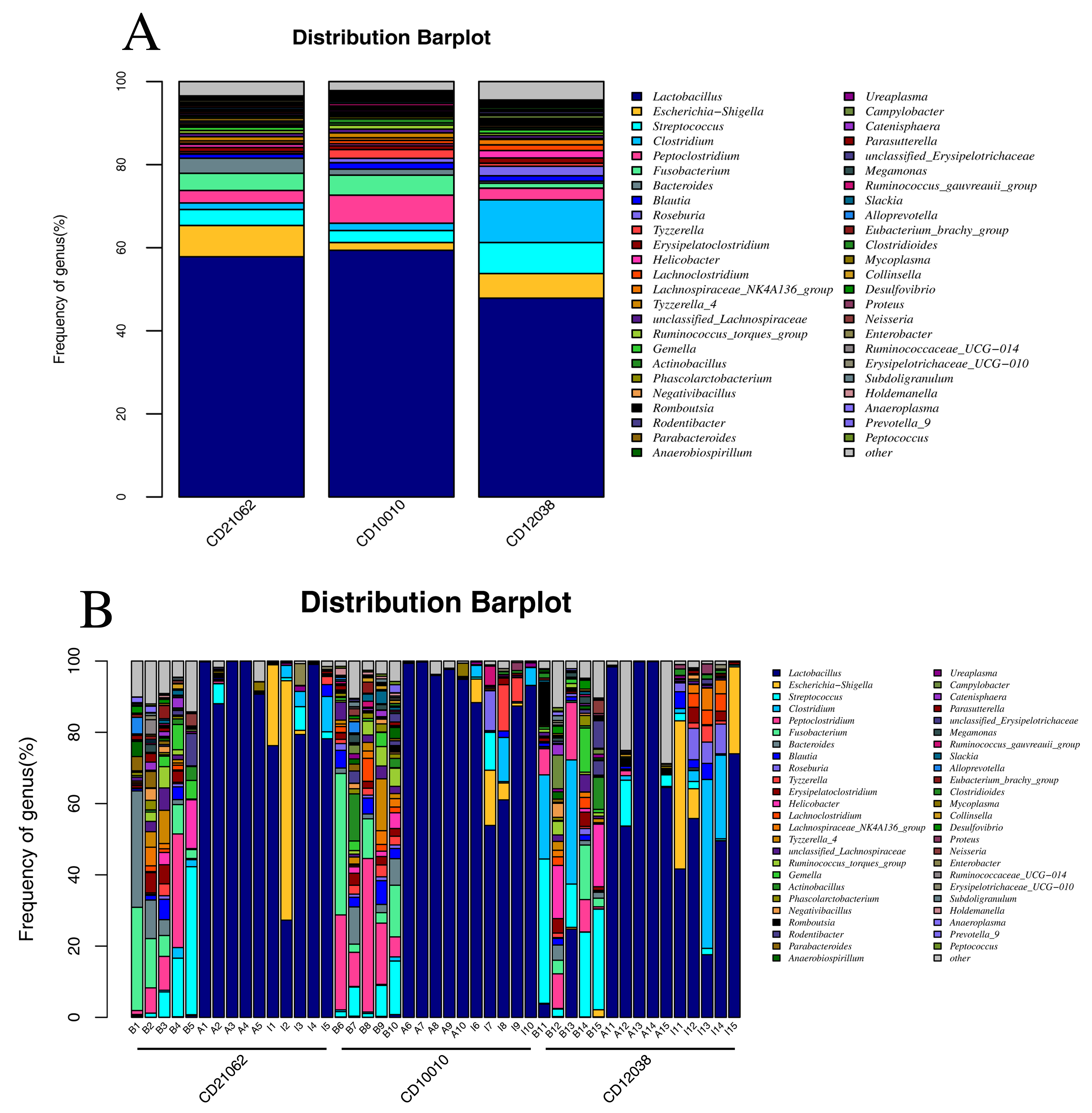

Supplement: Supplementary_figure_S5.tif [file TEMI_A_1900748_SM1178.tif]

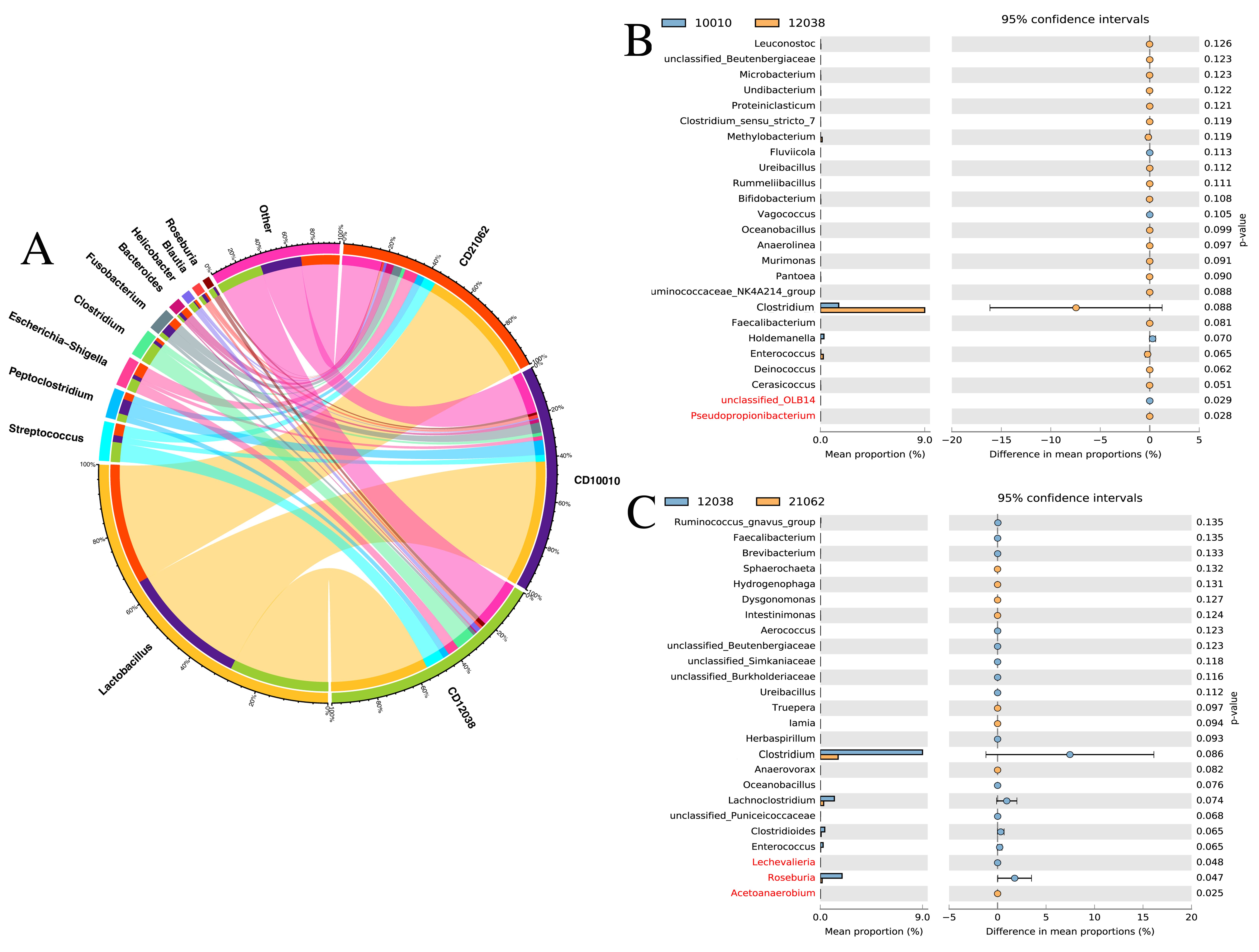

Supplement: Supplementary_figure_S4.tif [file TEMI_A_1900748_SM1177.tif]

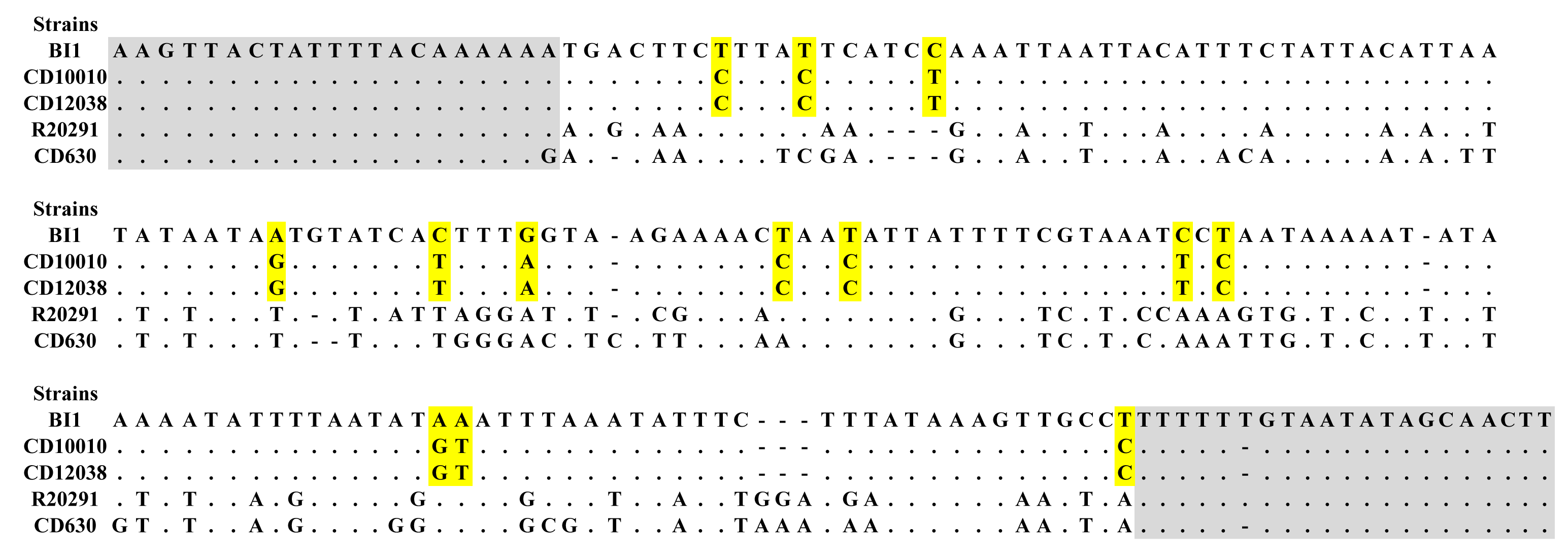

Supplement: Supplementary_figure_S3.tif [file TEMI_A_1900748_SM1176.tif]

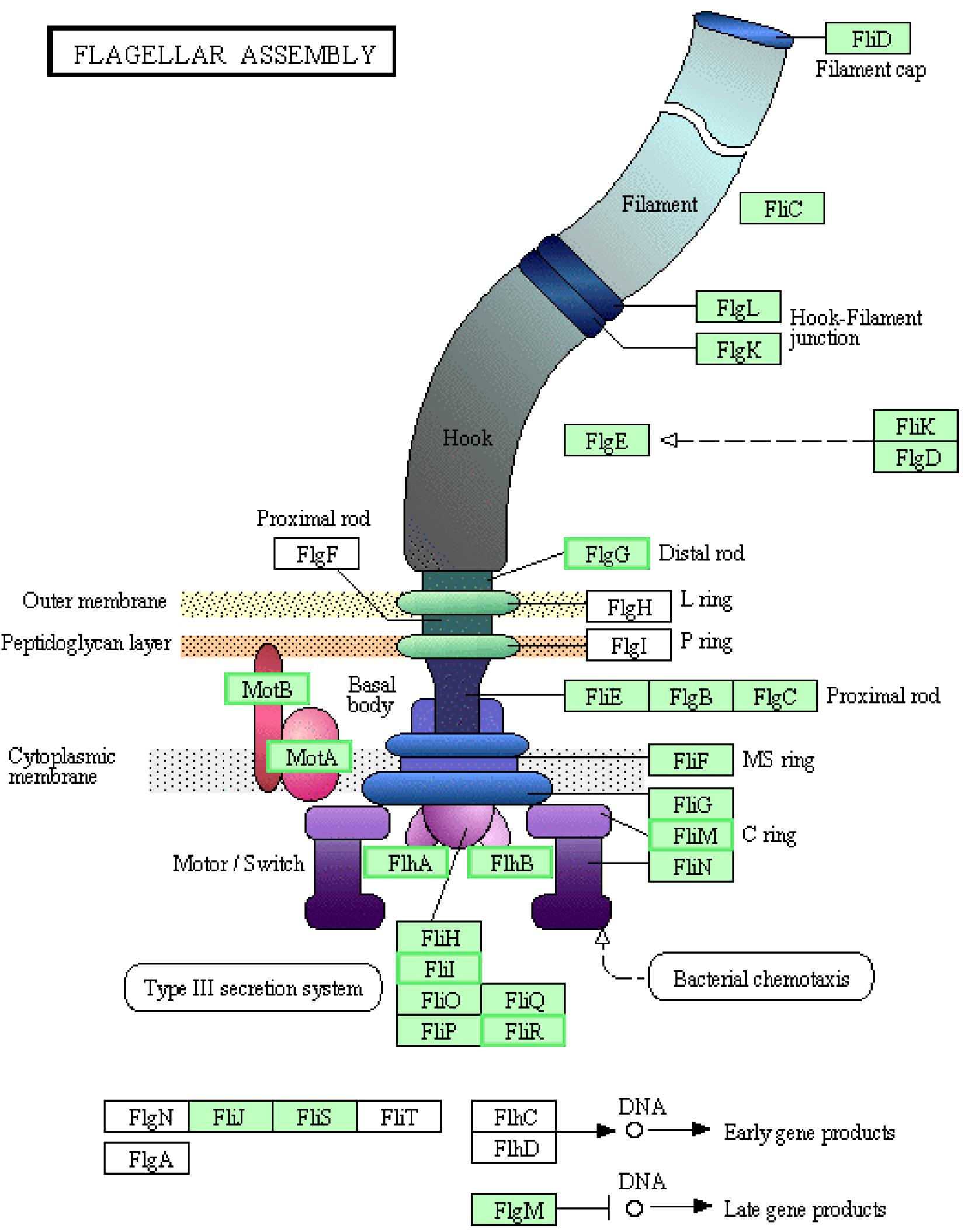

Supplement: Supplementary_figure_S2.tif [file TEMI_A_1900748_SM1175.tif]

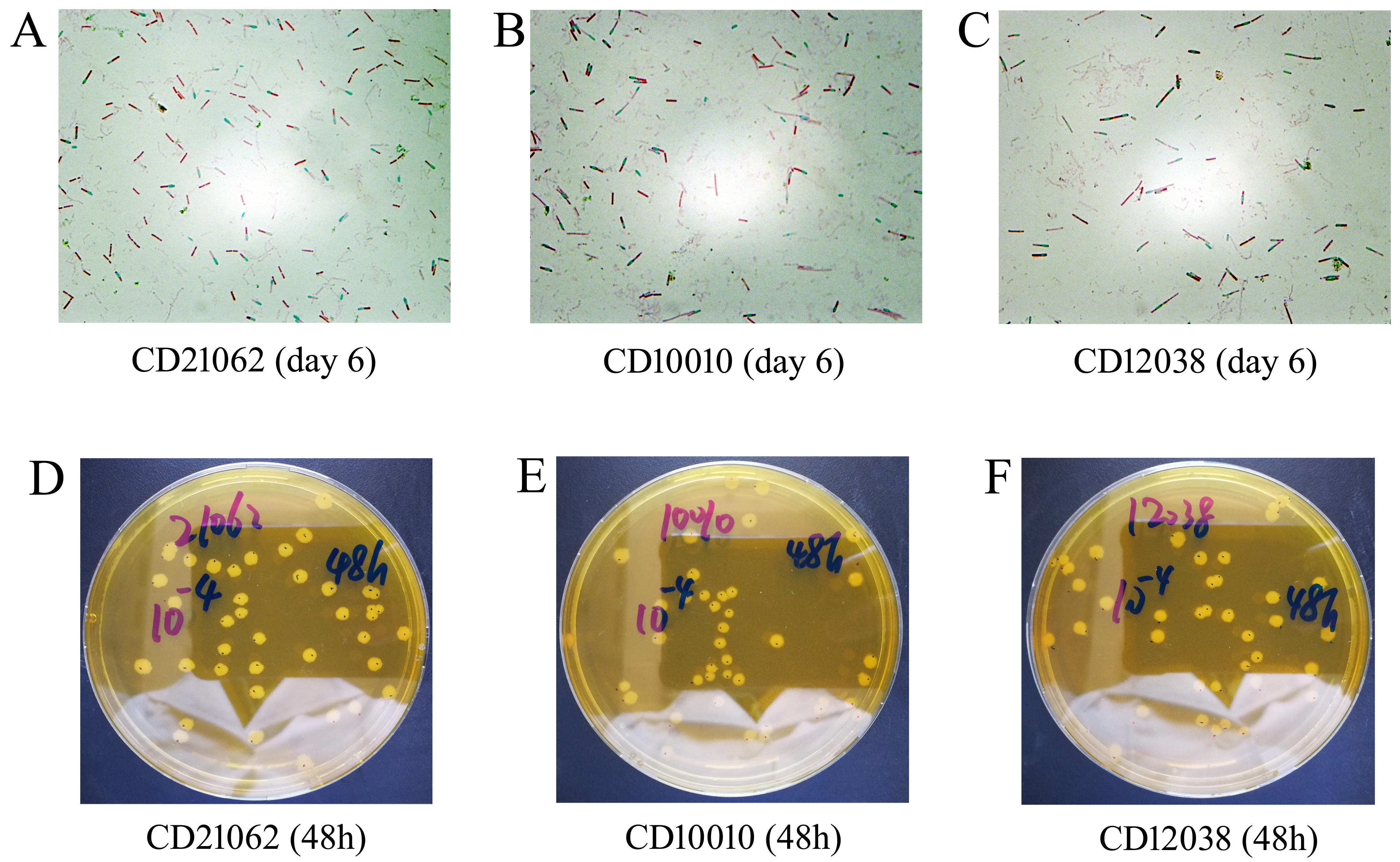

Supplement: Supplementary_figure_S1.tif [file TEMI_A_1900748_SM1174.tif]
